# Determinants of household decision making autonomy among rural married women based on Ethiopian demography health survey: a multilevel analysis

**DOI:** 10.1186/s12905-024-03058-3

**Published:** 2024-04-03

**Authors:** Desalegn Anmut Bitew, Amare Belete Getahun, Getachew Muluye Gedef, Fantahun Andualem, Mihret Getnet

**Affiliations:** 1 Department of Reproductive Health, Institute of Public Health, College of Medicine and Health Sciences, University of Gondar, Gondar, Ethiopia; 2 Department of Anesthesia, School of Medicine, College of Medicine and Health Sciences, University of Gondar, Gondar, Ethiopia; 3 School of midwifery, College of Medicine and Health Sciences, University of Gondar, Gondar, Ethiopia; 4 Department of psychiatry, School of Medicine, College of Medicine and Health Sciences, University of Gondar, Gondar, Ethiopia; 5 Department of Human Physiology, School of Medicine, College of Medicine and Health Sciences, University of Gondar, Gondar, Ethiopia

**Keywords:** Decision making, Household, Married women, Multilevel

## Abstract

**Introduction:**

Decisions made at the household level have great impact on the welfare of the individual, the local community, as well as the welfare of the nation. Women’s independent decision on reproductive health increases women’s access to health information and utilization of reproductive services. This has great impact on maternal and child health outcomes. However, women in developing or low-income countries often have limited autonomy and control over their household decisions. Therefore the main purpose of this research project is to investigate the potential determinants of rural women’s household decision making autonomy.

**Methods:**

A multi level analysis was performed using the fourth Ethiopian Demographic and Health Survey (EDHS) 2016 data set. A weighted sample of 8,565 married rural women was included in the final analysis. Women were considered to be autonomous if they made decisions alone or jointly with their husband in all three household decision components. It was dichotomized as yes = 1 and no = 0. Multico linearity and chi-square tests were checked and variables which did not fulfill the assumptions were excluded from the analysis. Four models were fitted. Variables with p-value ≤ 0.25 in the bi-variable multilevel logistic regression were included in the multivariable multilevel logistic regression. The Adjusted Odds Ratio (AOR) with a 95% confidence interval (95% CI) was computed. Variables with a P-value of less than 0.05 in the multi-variable multilevel logistic regression were declared as statistically significant predictors.

**Result:**

A total of 8,565 weighted participants involved. From the total respondents, 68.55**%** (CI: 67.5%, 69.5%) of women had decision making autonomy. wealth index (poor: AOR: 0.84; 95% CI: 0.72, 0.97 and middle: AOR: 0.85; 95% CI 0.73, 0.98), literacy (illiterate: AOR: 0.75; 95% CI: 0.66, 0.86), respondents working status (Not working; AOR 0.68; 95% CI; 0.60, 0.76) ,who decides on marriage (parents: AOR 0.76; 95% CI; 0.67, 0.87), and proportion of early marriage in the community (high proportion of early marriage AOR: 1.35; 95% CI; 1.10, 1.72).

**Conclusion:**

Women decision making autonomy was significantly determined by women economic participation (their wealth and their working status), women’s literacy, proportion of early marriage in the community and women’s involvement in decision of their marriage. Improving women’s economic participation and enhancing women’s participation to decide on their marriage will enhance women’s decision making autonomy.

## Introduction

Empowering women means capacitating them with any tools they need to have power and control over their own lives. Empowered women have freedom, equal opportunities, and the ability to makes choices in all areas of their lives [1]. Women’s Empowerment is a process by which individuals get power, develop confidence, increase awareness, improve control over resources, and make decisions [2].

Women’s decision-making autonomy is women’s ability to decide independently on their concerns [3]. Women’s independent decision on reproductive health issues is crucial for better maternal and child health outcomes; however, restriction of open discussion and decision limits women’s access to reproductive health services [4]. Women’s autonomy on the decision regarding health increases women’s access to health information and utilization of reproductive services [5].

Limited women’s autonomy prevents mothers from using maternal healthcare services such as, ante natal care (ANC), postnatal care (PNC), and delivery at a facility. Thus, Strong women’s decision-making power is essential to reduce maternal and child mortality and morbidity [6]. Lesser decision-making power of women negatively affect the fertility decision, usage of contraception, and sexual lives of women [7, 8]. Decisions made at the household level have great impact on the welfare of the individual, the local community, as well as the welfare of the nation [9, 10].

Women’s decision making power was significantly affected by age of respondents [11], respondent’s educational attainment [11–13], occupation [12, 13], income [11–13], and gender-based awareness [12, 13], and justification of wife-beating [13].

Efforts are being made by the international community to increase women’s access to decision-making. This is evidenced by one of the Sustainable Development Goals (SDGs), which stated as establishing gender equality and empowering all women and girls [14]. Ethiopia had the legal frame works that promote, enforce and monitor gender equality under SDG indicators with a focus on violence against [15] and has implemented affirmative action a constitutional laws and national legislatures that fosters women Empowerment [16]. However, in practice, women are still second class citizens in which 40.3% of women aged 20–24 years old were married or in union before age 18, the adolescent birth rate was 79.5 per 1,000 women aged 15–19. In 2018, 26.5% of women aged 15–49 years reported that they had been subjected to physical and /or sexual violence by current of former intimate partner [15, 16]. Even if women’s participation in a decision making will increase the uptake of healthcare services, facilitate poverty reduction, and enhance household economic growth, evidences suggest that women in developing or low-income countries often have limited autonomy and control over their household decisions [9, 11, 17, 18]. The UN government speculates the following triple mandates as a priority agenda to empower women both in developed and developing countries [19].

1. Promote coordination across the UN system to enhance accountability and results for gender equality and women’s empowerment;
2. Support UN Member States to strengthen global norms and standards for gender equality and women’s empowerment, and to include a gender perspective when advancing other issues; and.
3. Undertake operational activities at the country and regional levels, including supporting Member States in developing and implementing gender-responsive laws, policies and strategies that take into account women’s lived realities.

As far as our knowledge, prior researches considering the three main areas of decision making autonomy in the household (decision on the woman’s own health care, major household purchases, and visits to the woman’s family or relatives) among rural women in Ethiopia are limited. It is very crucial to identify the determinants of the decision making autonomy. Because, as directed by UN, developing and implementing gender-responsive laws, policies and strategies that take into account women’s lived realities is a priory agenda [19]. This study will provide inputs for this action therefore the main purpose of this research project is to investigate potential determinants of rural women’s household decision making autonomy. The finding from this study will provide an input for policy makers, program designers and project managers to design appropriate interventions incorporating the factors affecting the rural women’s participation on household decision making autonomy in the whole process of project design and program implementations.

## Methods

### Study setting, study design, period and sampling

This study was conducted in Ethiopia using the fourth Ethiopian demography and health survey (EDHS).

The sampling procedure in EDHS was a stratified, two stages. Each region was stratified into urban and rural areas. Stratification and proportional allocationwere performed at each lower administrative level by sorting the sampling frame within each sampling stratum. Data collection took place over data collection took place over a 5.5 month period, from January 18, 2016, to June 27, 2016. The detailed sampling method has been explained in the methodology section of EDHS report [20].

### Data source and study population

We have used individual record (IR) data set of EDHS 2016 for this study. The data was accessed from the measure DHS website (http://www.measuredhs.com). Interviews were done for 15,683 women of reproductive age across urban and rural strata, of whom, 9,824 were already married (currently living with husband or partner). Of those, 2,491 were urban residents and 7,333 were rural residents. The current study includes only rural married women. After weighting, a total of 8,565 rural married women were included in the final analysis. All the frequencies and percentages in the result section were weighted.

### Variables and measurements

The outcome variable in this study was women’s decision making autonomy in the house hold. Women were asked the following three questions.

1. Who decided on women own health care?
2. Who decided on major household purchases? and.
3. Who decided on visits to the woman’s family or relatives?

Women were considered to be autonomous if they made decisions alone or jointly with their husband in all three of the above questions. Other ways they were considered not autonomous [20]. It was dichotomized as (yes = 1 and no = 0). The final aggregate measure of Women’s decision making autonomy was computed from the three major components of household decision making (decision on visits to family or relatives, decision on respondent’s health care and decision on large household purchases). First, the three components were dichotomized as “Yes” if a woman decides jointly or alone and “No” if a woman didn’t decide. We generate the outcome variable by adding the three components. Finally the intersection of the three was considered as “Yes”.

The independent variables were socio-demographic and husband related characteristics such as age, educational level, wealth index, literacy, religion, media exposure, decision on marriage, age at first sex, respondents working status, husband education, husband working status, sex of household head and age of household head and health insurance coverage. As well as community level variables include community wealth, community education, and community proportion of early marriage, community literacy and community media exposure.

### Individual level variables

**Educational status of women**: This variable was divided into four categories: no education primary, secondary and higher education.

#### Wealth index

In the dataset, the wealth index was categorized as Poorest, Poorer, Middle, Richer, and Richest. In this study, a new variable was generated with three categories as “Poor”, “Middle” and “Rich” by merging poorest with poorer and richest with richer.

#### Religion

In the data set, religion was categorized as Orthodox, Muslim, Protestant, Catholic, traditional followers and others. In this study, the former three were encoded independently and Catholic and traditional religion followers were merged into the “others” category.

#### Working status

this has been categorized as “Yes” and “No” in the 2016 EDHS.

#### Media exposure

Watching television (TV), listening to the radio and reading newspapers both less than once a week and at least once a week were considered to measure exposure to media.

#### Age at first sex

Was categorized into four as “never had sex, “active before age 15,” “active between ages 15 and 17,” and “active at age 18 and above”.

### Community level variables

Community-level variables were computed by aggregating the individual level women’s characteristics into clusters. Then the proportion was calculated by dividing subcategories by the total. Distributions of the proportion of aggregate variables were checked using the Shapiro–Wilk normality test and were not normally distributed. Therefore, these aggregate variables were categorized using the median value. Five community level variables were generated.

### Data processing and analysis

Descriptive statistics such as frequencies and percentages were computed once the data had been cleaned. We used Stata soft ware to analyze the data. Sampling weights were used to account for the sample’s non-proportional strata allocation and non-responses. Individuals were nested inside communities in the EDHS data, and the intra-class correlation coefficient (ICC) was 20.50%. Before fitting the model, we tested the chi square assumption. As a result, early marriage, husband working status, husband education and respondents age were failed to fulfill the chi square assumption and were excluded from the model. Multi-co linearity was also checked and variance inflation factor (VIF) for respondent’s educational status was greater than 10 and we excluded it from the regression. To evaluate the independent (fixed) effects of the explanatory variables as well as the community-level random effects on the outcome variable, a two-level mixed-effects logistic regression model was fitted. We fitted four models (Null Model (no factors), Model 1 (0nly individual level factors), Model 2 (only community-level factors), and Model 3 (both individual and community-level factors)). Variables with a p-value of < = 0.25 from the bi-variable multilevel logistic regression analysis were included in the multivariable multilevel logistic regression analysis. The Adjusted Odds Ratio (AOR) with a 95% confidence interval (95% CI) was computed. Variables with a P-value of less than 0.05 in the multi-variable multilevel logistic regression analysis in the final model were declared as statistically significant determinants of women’s decision making autonomy.

## Result

### Individual level characteristics of respondents

The median age and the mean age of respondents was 30 years and 30.60 (± 8.30) respectively. Totally, 8565 married rural women participated in this study. About 69% of respondents had no formal education. Nearly half (46.18%) of the respondents were from poor socio economic class. About three forth (76.10%) of respondents had early marriage. The decision of marriage was made by parents for 64.68% of respondents (Table 1).

**Table 1:** Individual level characteristics and women’s decision making autonomy (*n* = 8565), Ethiopia

Variables	Categories	Frequency (%)	Women’s decision making autonomy (%)	
			No (%)	Yes (%)
Educational status	No education	5,880 (68.66)	1,905 (22.24)	3,975 (46.42)
	Primary	2,354 (27.48)	722 (8.43)	1632 (19.05)
	Secondary	270 (3.15)	61 (0.72)	209 (2.4)
	Higher	61 (0.71)	6 (0.06)	55 (0.65)
Husband education	No education	4,500 (52.54)	1415 (16.53)	3084 (36.01)
	Primary	3,301 (38.54)	1047 (12.22)	2254 (26.32)
	Secondary	544 (6.35)	181 (2.12)	363 (4.23)
	Higher	220 (2.58)	50 (0.59)	170 (1.99)
Literacy	Literate	1,801 (21.03)	451 (5.26)	1350 (15.77)
	illiterate	6,764 (78.97)	2243 (26.19)	4521 (52.78)
Wealth index	Poor	3,955 (46.18)	1348 (15.74)	2607 (30.43)
	Middle	2,035 (23.76)	664 (7.75)	1371 (16.01)
	Rich	2,575 (30.07)	681 (7.96)	1894 (22.11)
Religion	Orthodox	3,174 (37.05)	870 (10.15)	2304 (26.9)
	Muslim	3,179 (37.13)	1037 (12.11)	2142 (25.02)
	Protestant	1,979 ( 23.10)	693 (8.09)	1286 (15.02)
	Others ^a^	233 (2.72)	94 (1.1)	139 (1.62)
Working status	Working	3,872 (45.20)	1053 (12.29)	2819 (32.91)
	Not working	4,693 (54.80)	1641 (19.15)	3053 (35.64)
Early marriage	Yes	6,518 (76.10)	2028 (23.68)	4490 (52.42)
	No	2,047 (23.90)	666 (7.77)	1381 (16.13)
Age at first sex	< 15	2,120 (24.75)	600 (7.00)	1520 (17.75)
	15–17	3,866 (45.14)	1266 (14.79)	2600 (30.35)
	>=18	2,579 (30.11)	827 (9.66)	1752 (20.45)
Decision maker for marriage	My self	2,652 (30.96)	764 (8.92)	1888 (22.05)
	Parents	5,540 (64.68)	1805 (21.07)	3735 (43.61)
	Other relatives	373 (4.36)	125 (1.46)	248 (2.90)
Media exposure	Yes	6,955 (81.20)	2216 (25.88)	4739 (55.32)
	No	1,610 (18.80)	477 (5.57)	1133 (13.23)
Health insurance	Yes	8,161 (95.28)	2584 (30.17)	5577 (65.11)
	No	404 (4.72)	110 (1.28 )	294 (3.44)
Sex of HHH	Male	7,625 (89.02)	2435 (28.43)	5190 (60.6)
	Female	940 (10.98)	259 (3.02)	681 (7.95)

Key: HHH: Household head: Others ^a =^catholic, traditional and others

### Community level characteristics of the respondents

Five thousands and nine hundreds twenty nine (69.23%) of respondents were from a community with low proportion of poorness. Nearly half (49.77%) of respondent were from a community with high proportion of no education (Table 2).

**Table 2:** Community level characteristics and Women’s decision making autonomy (*n* = 8565), Ethiopia

Variables	Categories	Frequency (%)	Women’s decision making autonomy (%)	
			No (%)	Yes (%)
Community wealth	Low proportion of poor	5,929 (69.23)	1803 (21.05)	4127 (48.18)
	High proportion of poor	2,636 (30.77)	891 (10.4)	1745 (20.37)
Community education	Low proportion No education	4,302 (50.23)	1315 (15.35)	2987 (34.88)
	High proportion no educated	4,263 (49.77)	1379 (16.1)	2884 (33.67)
Community early marriage	Low proportion of marriage	4,480 (52.30)	1501 (17.52)	2979 (34.78)
	High proportion of marriage	4,085 (47.70)	1193 (13.93)	2892 (33.77)
Community literacy	Low proportion of literate	3,572 (41.70)	1207(14.1)	2365 (27.61)
	High proportion of literate	4,993 (58.30)	1486 (17.35)	3507 (40.94)
Community media exposure	Low proportion of media exposure	3,784 (44.18)	1234 (14.41)	2550 (29.77)
	High proportion of media exposure	4,781 (55.82)	1,459 (17.04)	3,321 (38.78)

### Model selection

Multilevel mixed effect logistic regression model was fitted. The measures of variations or random effects were reported using intra-class correlation (ICC), a proportional change in variance (PCV), and Median Odds Ratio (MOR). The ICC was used to show how much the observation within one cluster resembled each other and it was generated directly from each model using “estat ICC “command following regression. PCV was computed using the following formula.

$PCV=\frac{Vnull -VA}{V null }$ [21] and MOR was computed to measure unexplained cluster heterogeneity and it was calculated using the formula $:MOR={e}^{0.95\sqrt{VA}}$ [21] where “VA” represents the area or cluster level variance for each model. The model comparison was done using Akaike’s information criterion (AIC). The model with smallest AIC was selected. Therefore, model III was the best fit model with AIC 9819.714 (Table 3**).**

**Table 3:** Random effect and two-level mixed effect logistic regression models predicting Women’s decision making autonomy, Ethiopia

Parameters	Null model	Model III (final model)
Community Variance (SE)	0.87 (0.10)	0.84 (0.10)
AIC	9913.297	9819.714
BIC	9927.097	9957.716
ICC	21%	20.5%
PCV	Reference	3.45%
MOR	2.41	2.36
Log likelihood	-4954.64	-4876.15

Key: ICC: intra class correlation, PCV: proportional change in variance and MOR: Median Odds Ratio

Magnitude of Women’s decision making autonomy from the total respondents, 68.55**% (CI: 67.5%, 69.5%)** women’s had decision making autonomy. Women’s participation on visits to family or relatives, respondent’s health care and large household purchases were 82.23%, 79.57% and 76.29% respectively (Table 4).

**Table 4:** The magnitude of Women’s decision making autonomy among married women in the study of Women’s decision making autonomy and determinants (*N* = 8,565: weighted), Ethiopia

Component of decision	Category	Frequency (%)
Decides on respondent’ s health care	Yes	6,815 (79.57)
	No	1,750 ( 20.43)
Decides on visits to family or relatives	Yes	7,043 (82.23)
	No	1,522 (17.77)
Decides on large household purchases	Yes	6,534(76.29)
	No	2,031 (23.71)
Over all decision making autonomy.	Yes	5,871( 68.55)
	No	2,694 (31.45)

### Determinants of women decision making autonomy (WDMA)

In this study wealth, working status, literacy and decision on marriage from individual level factors and proportion of early marriage from community level factors were statistically significant predictors of **WDMA**.

The odds of **WDMA** among poor women and Middle class women was reduced by 16% (poor: AOR: 0.84; 95% CI: 0.72, 0.97) and 15% (middle: AOR: 0.85; 95% CI 0.73, 0.98) respectively compared to rich women. The odds of **WDMA** illiterate women was reduced by 25% (Illiterate: AOR: 0.75; 95% CI: 0.66, 0.86) compared to literate women. The odds of **WDMA** among women who didn’t work was reduced by 32% (Not working; AOR 0.68; 95% CI; 0.60, 0.76) compared to women who were working.

The odds of **WDMA** among women whose marriage was decided by their parents was reduced by 24% (parents: AOR 0.76; 95% CI; 0.67, 0.87) compared to women who decided by themselves. The odds of **WDMA** among women from a community with high proportion of early marriage were increased by 35% (high proportion of early marriage; AOR: 1.35; 95% CI; **1.10, 1.72**) compared to women from a community with low proportion of early marriage (Table 5).

**Table 5:** Individual and community-level factors associated with women decision making autonomy (**WDMA**) (*n* = 8565), Ethiopia

Individual and Community Level Characteristics		COR (95% CI)	Final Model AOR (95% CI)
Religion	Orthodox	Ref	Ref
	Muslim	0.83 (0.68,1.03)	0.99 (0.79, 1.23)
	Protestant	0.89 (0.71, 1.10)	0.90 (0.72, 1.13)
	Others^*^	0.81 (0.53,1.21)	0.84 (0.56, 1.27)
Wealth	Poor	0.77(0.67,0.89)	**0.84 (0.72, 0.97)** ^*^
	Middle	0.81(0.70, 0.94)	0.85 (0.73, 0.98)*
	Rich	Ref	Ref
Literacy	Literate	Ref	Ref
	illiterate	0.69(0.60,0.79)	0.75 (0.66, 0.86)**
Age at first sex	< 15	1.05 (0.91,1.22)	1.12 (0.97, 1.31)
	15–17	0.91 (0.81,1.03)	0.93 (0.83, 1.05)
	>=18	Ref	Ref
Decision maker for marriage	My self	Ref	Ref
	Parents	0.76 (0.67,0.86)	0.76 (0.67, 0.87)**
	Other relatives	0.87 (0.67,1.11)	0.84 (0.65, 1.09)
Working status	Working	Ref	Ref
	Not working	0.67 (0.59,0.75)	0.68 (0.60, 0.76)**
Media exposure	Yes	Ref	Ref
	No	0.87 (0.76,0.99)	0.97 (0.84,1.11)
Health insurance	Yes	Ref	Ref
	No	1.07 (0.81,1.42)	1.16 (0.87, 1.54)
Community wealth	Low proportion of poor	Ref	Ref
	High proportion of poor	0.72 (0.57, 0.92)	1.18 (0.89, 1.56)
Community education	Low proportion No education	1.19 (0.94 ,1.52)	0.93 (0.70, 1.24)
	High proportion no education	Ref	Ref
Community early marriage	Low proportion of marriage	Ref	Ref
	High proportion of marriage	1.24 (0.98 ,1.58)	1.35 (1.10, 1.72)*
Community literacy	Low proportion of literate	0.67 (0.53,0.86)	0.75 (0.56,1.01)
	High proportion of literate	Ref	Ref
Community media exposure	Low proportion of media exposure	0.88 (0.69,1.12)	1.09 (0.84, 1.42)
	High proportion of media exposure	Ref	Ref

Key: Ref = Reference, *=significant at p value < 0.001 and **= significant at p value < 0.05

## Discussion

We investigated the potential determinants rural women decision making autonomy. As a result, women’s wealth index, their working status, their literacy, their involvement in their marital decision and high proportion of early marriage in the community was found to be significantly associated with WDMA.

Our study showed that women with lower household wealth indexes had lower decision making autonomy. This finding is supported by findings from, Ghana [22], Burkina Faso [23] and Nepal [4], women from wealthier households were more likely to participate in decision-making, either jointly or individually. This may be explained by women in poor household are likely to be uneducated and they may lack the knowledge and skill of negotiating decision. Their economic condition might also limit women’s purchasing power. This is because the ownership and control of property had great impact on minimizing gender gap and enhance economic wellbeing, social status, and empowerment [12, 24]. This implies that programs and projects policies and strategies designed to empower should give special attention for women in low socio economic classes.

Literacy was also positively associated with women decision making autonomy. This shows literate mothers had increased odds of decision making autonomy compared to illiterate mothers. This was supported by other findings from Ghana [22] and Nepal [4]. Most of literate mothers had at least primary and above educational attainment. In our study, about 92% of literate mothers had primary and above educational attainment. Evidences showed that, Women who had higher educational attainment had higher decision making autonomy. Because, education improve their knowledge, negotiating abilities, and self-confidence [25–27], improves employment chances [27–29], and reduces the occurrence gender-based violence [22, 30, 31]. `This implies that increasing women’s literacy through different mechanisms like increasing their enrollment to either traditional or formal education and increasing their attainment to higher level of education should be mainstreamed by ministry of education and other program managers.

This study also revealed that women working status had significant association with their autonomy in decision making. This finding was supported by findings from Burkina Faso [23] and Nepal [4] which showed that Women’s participation in household decisions is enhanced while they are working. This is due to the fact that women who are working will have capacity to afford costs related to their own health care as well as other major purchases which in turn improves women’s participation in decision making regarding their own health care, household purchases or visiting family or friends [4, 23]. This finding implies that ministry of labor and skills of Ethiopia in collaboration with other stake holders like civil services, Nongovernmental organization and institutions should facilitate job opportunities for women.

Surprisingly, our study revealed that women who were form a community with high proportion of early marriage had increased odds of decision making autonomy. This was supported by one evidence from Bangladesh [32] which stated that, the autonomy level of women who got married in their earlier age have the highest level of autonomy in all three dimensions of house hold decisions. This may be explained by the fact that, the formation of first marriage brings important changes in a women’s family situation and in thier future expectations and opportunities. Marriage is the time when couples start their own life independent of their family [32]. However, this finding contradicted with other findings from Ethiopia [33, 34] and Indonesia [35]. This contradiction may be due to the difference in the study population and the difference in the operational definition for the outcome ascertainment as well as the difference the interest of the outcome. For example in our study, the populations were all married rural women where most of the household tasks are given for women. Where as in the previous study, the population were all married women regardless of their residency [33, 34]. In the previous study the outcome was decision regarding to contraception and women was considered autonomous if the decide independently [33, 34], while in the current study, the outcome was decision making autonomy for the three major household decisions (own health care, household purchase and family visit) and women were considered autonomy if they make decision alone or jointly for all the three components of household decisions. The other possible explanation for this contradiction can be the nature of the outcome variable. It is obvious that decision on contraceptive use is somehow sensitive than decisions on household purchase and family visit. This contradictory finding revealed that researchers should further investigate the reasons using advanced research designs like prospective cohort and qualitative research design.

Another important determinant of women’s decision making autonomy was their power of decision on their marriage. In this study, women whose marriage was decided by their parents had reduced odds of decision making autonomy. This study finding was supported by finding from Pakistan [36]. This could be explained women who are able to express their opinion and are part of the decision for their own marriage, they might be confident in communicating and negotiating with their husband once married. This implied that, the involvement of women in their marital decision will enhance their decision making autonomy in the household.

## Conclusion

Generally women’s decision making autonomy was high (68.55%) compared to other developing countries [37]. Women decision making autonomy was significantly determined by women economic participation (their wealth and their working status), women’s literacy, proportion of early marriage in the community and their participation in their marriage. Improving women’s economic participation and enhancing women’s participation to decide on their marriage will enhance women’s decision making autonomy. Qualitative researches should also be conducted to explore reasons for the contradictory findings (Does early age marriage positively associated with women’s decision making autonomy? ).

### Strengths and limitations

As strength, we used nationwide data which increased the representativeness of the finding and we used advanced statistical model which solved the effect hierarchal nature of the data set. On the other hand, using secondary data limit the researcher to measure all possible factors such as culture and tradition-related factors as well as the individuals perception on the severity of the illness for health care decision. The source of the data was self-report which affect the accuracy of the data by recall bias. The data for this conclusion was from cross-sectional survey and it does not show causality.

## Data Availability

The dataset supporting the conclusions of this article were accessed through request on the measure DHS website (http://www.measuredhs.com) and the extracted data used during the current analysis is available from the corresponding author on reasonable request.

## Abbreviations

AIC: Akaike’s: Information Criterion: Ante Natal Care
AOR: Adjusted Odds Ratio
BIC: Bayesian Information Criterion
CI: Confidence interval
COR: Crude Odds ratio
EDHS: Ethiopia Demographic and Health Survey
ICC: Intra Class Correlation
MOR: Median Odds Ratio
PCV: Proportional Change in Variance and WDMA: Women Decision Making Autonomy
